# Getting to the heart of sarcopenia and cardiovascular disease

**DOI:** 10.18632/aging.206296

**Published:** 2025-08-05

**Authors:** Pouya Nezafati, Pankaj Saxena, Jaishankar Raman, Lionel Hebbard, Craig McFarlane

**Affiliations:** 1 Discipline of Biomedical Sciences and Molecular Biology, College of Medicine and Dentistry, Australian Institute of Tropical Medicine and Health, Centre for Molecular Therapeutics, James Cook University, Townsville, QLD 4811, Australia; 2 Department of Cardiothoracic Surgery, John Hunter Hospital, Newcastle, New South Wales 2305, Australia; 3 College of Medicine and Dentistry, James Cook University, Townsville, QLD 4811, Australia; 4 Department of Cardiothoracic Surgery, Townsville University Hospital, Townsville, Queensland 4810, Australia

**Keywords:** sarcopenia, cardiovascular disease, cardiac surgery, screening, diagnosis

## Abstract

Sarcopenia and cardiovascular disease are reported to have similar pathophysiological mechanisms, which suggests that management strategies for one could reduce the burden of the other. A variety of biomarkers linked to changes in the neuromuscular junction, endocrine systems as well as growth factors and muscle proteins have been utilised to assess and identify patients with sarcopenia or patients at risk of developing sarcopenia. Sarcopenia working groups have defined a set of consensus clinical guidelines to diagnose sarcopenia which have been commonly adopted globally. In addition, screening questionnaires have been introduced with different degrees of sensitivity and specificity to identify patients at risk of sarcopenia. Herein, we review screening and diagnosing strategies of sarcopenia as well as the different imaging modalities to quantify sarcopenia. The association of sarcopenia and cardiovascular disease are explored in terms of pathophysiological mechanisms. The effects of sarcopenia on cardiac surgery are evaluated, along with different preventive measures and treatment plans for sarcopenia.

## INTRODUCTION

Collectively, the group of disorders classified as cardiovascular disease (CVD) are the foremost cause of death worldwide [1]. A staggering 19.8 million deaths were attributed to CVD in 2022 [2], which equates to approximately 1/3 of global deaths annually [3]. Moreover, the prevalence of CVD is expected to rise [4], which can be attributed to a global aging population and increasing risk factors, including environmental (e.g. pollution), metabolic (e.g. blood pressure, diabetes) and behavioural (e.g. exercise, alcohol, smoking) [2]. Therefore, it is crucial to implement prevention and management strategies to alleviate the burden of CVD. While the contribution of various risk factors to different aspects of CVD has been extensively studied, sarcopenia has emerged as a significant comorbidity of CVD [5]. Importantly, the presence of CVD and its risk factors, such as obesity, insulin resistance, and inflammation, significantly increases the likelihood of developing sarcopenia [5, 6]. Furthermore, studies have revealed a close link between sarcopenia, overall metabolic dysfunction and an increased risk of CVD [5]. Clinically, sarcopenia and the presence of other CVD risk factors are associated with poorer health and worsened surgical outcomes for CVD patients [7, 8]. Therefore, it is crucial to identify and intervene early in cases of sarcopenia to effectively manage and prevent the progression and clinical consequences of this disease.

## Definition of sarcopenia

The term Sarcopenia was initially introduced to describe the progressive loss of skeletal muscle mass typically observed during aging [9]. This definition has expanded to include reduced muscle strength (function) and impaired physical performance [10]. In 2016, sarcopenia was classified as a disorder of muscle and given the code M62.84 under the international classification of disease, tenth revision clinical modification (ICD-10-CM) system [11].

## Clinical diagnostic guidelines

Several working groups have described consensus guidelines for testing and diagnosing sarcopenia in the clinic. These studies have proposed several population-specific cut off values for a group of important clinical tests for sarcopenia, which are summarized in Table 1.

**Table 1:** Common clinical diagnosis guidelines of sarcopenia.

**Classification**	**Definition**	**Muscle mass**	**Muscle strength**	**Physical performance**
**EWGSOP (2010) [12]**	**Sarcopenia:** low muscle mass **and** low muscle strength **or** low physical performance	**DXA** ASM/height^2^ Men 7.26 kg/m^2^ Women <5.5 kg/m^2^ **BIA** SM/height^2^ Men <8.87 kg/m^2^ Women<6.42 kg/m^2^	Handgrip Strength Men <30 kg Women <20 kg	**Men and women** Gait speed <0.8 m/s SPPB ≤8
**EWGSOP2 2019 [13]**	**Probable Sarcopenia:** low muscle strength **Confirmed Sarcopenia:** low muscle mass **and** low muscle strength **Severe Sarcopenia:** low muscle mass, low muscle strength **and** low physical performance	**DXA** ASM/height^2^ Men <7.0 kg/m^2^ Women <5.5 kg/m^2^	Handgrip Strength Men <27 kg Women <16 kg Chair stand >15s for 5 chair rises	**Men and women** Gait speed <0.8 m/s SPPB ≤8 TUG ≥20s 400m walk test, failure to finish or finish in ≥6 min
**AWGS 2014 [14]**	**Confirmed Sarcopenia:** low muscle mass with low muscle strength **and/or** low physical performance	**DXA** ASM/height^2^ Men 7.0 kg/m^2^ Women <5.4 kg/m^2^ **BIA** ASM/height^2^ Men <7.0 kg/m^2^ Women <5.7 kg/m^2^	Handgrip Strength Men <26 kg Women<18kg	**Men and women** Gait speed ≤0.8 m/s
**AWGS 2019 [15]**	**Possible Sarcopenia:** low muscle strength **or** low physical performance **Confirmed Sarcopenia:** low muscle mass **with** either low muscle strength **or** low physical performance **Severe Sarcopenia:** low muscle mass, low muscle strength, **and** low physical performance	**DXA** ASM/height^2^ Men 7.0 kg/m^2^ Women <5.4 kg/m^2^ **BIA** ASM/height^2^ Men <7.0 kg/m^2^ Women <5.7 kg/m^2^	Handgrip Strength Men <28 kg Women <18 kg	Gait speed <1.0 m/s SPPB ≤9 Chair stand >12s for 5 chair rises
**IWGS 2011 [16]**	**Confirmed Sarcopenia:** low muscle mass **and** low physical performance	**DXA** Men ≤7.23 kg/m^2^ Women ≤5.67kg/m^2^		**Men and women** Gait speed <1.0 m/s

Table summarising current commonly used guidelines for diagnosing sarcopenia. ASM: appendicular skeletal muscle mass; AWGS, Asian Working Group for Sarcopenia; BIA, bioelectrical impedance analysis; BMI, body mass index; CT, computed tomography; DXA, dual-energy x-ray absorptiometry; SPPB, Short Physical Performance Battery test; TUG, timed up and go test, which assesses the time taken to rise from a chair, walk a distance, return and sit down; SM, predicted skeletal muscle mass from BIA; EWGSOP, European Working Group on Sarcopenia in Older People; IWGS, International Working Group on Sarcopenia.

Initially in 2010, the European Working Group on Sarcopenia People (EWGSOP1) introduced a clinical guideline to diagnose sarcopenia as a syndrome, focusing on a progressive loss of skeletal muscle mass as well as function (physical performance or strength) (Table 1) [12]. The EWGSOP1 further suggested different stages for sarcopenia, consisting of pre-sarcopenia (decreased muscle mass, but no changes in strength or performance), sarcopenia (decreased muscle mass, with either decreased muscle strength or performance) and severe sarcopenia (decreased muscle mass, strength and performance) [12]. Skeletal muscle mass is typically determined by imaging, and function is generally measured using hand-grip strength and physical performance, evaluated through a gait speed test or SPPB (Short Physical Performance Battery test) which is a combined test consisting of gait speed, balance and chair stand assessments. SPPB is scored out of a possible maximum of 12 points (where 0-6 is considered low; 7-9 intermediate and 10-12 high performance) [12] (Table 1).

EWGSOP2 revised the definition in 2019 and introduced low muscle strength as the primary sarcopenia indicator, rather than loss of muscle mass [13]. Severe sarcopenia was defined as low muscle strength with decreased physical performance and pre-sarcopenia as decreased muscle strength alone (Table 1). Moreover, EWGSOP2 sub-categorized sarcopenia as acute or chronic, with acute sarcopenia categorised as lasting for <6 months and chronic if it is present for ≥6 months [13].

However, in 2014 the Asian Working Group for Sarcopenia (AWGS) [14] offered a sarcopenia definition that differed from the above, in requiring all the criteria to be considered, including low muscle mass, low physical performance and muscle strength (Table 1). Differing cut-off points were also proposed, based on findings from an Asian population group [14]. In 2019 the AWGS updated the definition of sarcopenia with different cut-off points proposed based on an Asian population, with diagnosis requiring loss of muscle mass in addition to decreased muscle strength and/or decreased physical performance (Table 1). Severe sarcopenia was considered when all criteria were present [15]. The AWGS also introduced “possible sarcopenia” as having low muscle strength or low physical performance. Moreover, the EWGSOP1, EWGSOP2, and AWGS Algorithms suggested that muscle mass be expressed relative to height^2^, termed the skeletal muscle index (SMI). The International Working Group on Sarcopenia (IWGS) considered low muscle mass and poor performance to diagnose sarcopenia (Table 1) and further defined a population of individuals that should be considered for sarcopenia and subsequent muscle mass assessment [16]. This includes all patients who are older and present with reduced strength, physical function or overall health, including bedridden patients, individuals who cannot rise from a chair unassisted and patients with a reduced gait speed (<1m/s) [16]. The Foundation for the National Institute of Health (FNIH) included muscle mass, physical and strength performance in the sarcopenia definition, recommending adjustments of muscle mass by BMI [17]. The Australian and New Zealand Society for Sarcopenia and Frailty Research (ANZSSFR) has suggested using the EWGSOP1 criteria, and population-specific cut-points [18].

Despite significant advancements in our understanding of sarcopenia, important limitations persist in the diagnostic strategies outlined above. One major challenge lies in the lack of consensus across diagnostic criteria, which vary substantially between international guidelines such as EWGSOP2, AWGS, and FNIH. This is critically important, as without a harmonised diagnostic framework, the inconsistencies in population-specific cut-offs for muscle mass, strength, and performance, will continue to complicate cross-study comparisons and hinder global implementation of standardized screening protocols.

## Prevalence of sarcopenia

The variation in diagnostic criteria and in the setting where studies have been performed, has led to discrepancies in reported prevalence rates globally, thus creating challenges in comparing results. A systematic analysis of previous studies has revealed the highest rates of sarcopenia in nursing homes, followed by hospitals, then in individuals in the general population [19]. Furthermore, it revealed a higher prevalence of sarcopenia in community-dwelling individuals from non-Asian countries when compared to Asian countries, specifically 13% versus 9% in men and 11% versus 8% in women, respectively [19].

These differences could be attributed to several factors, including genetic, cultural and environmental differences between these populations.

Significant differences in sarcopenia prevalence in community-dwelling adults have been noted between different populations, leading to a range of reported prevalence rates. Among Chinese adults who were at least 60 years of age, prevalence was reported to range between 21.6% in women to 19.2% in men [20]. In Japanese adults 60 years of age or older, the pooled prevalence rates from a systematic review were estimated to be between 9.8% for men and 10.1% for women [21]. One study revealed the prevalence of sarcopenia in Taiwanese adults aged over 65, to vary from 0.4% to 6.7% in women and men, respectively [22]. Whereas, a second study determined the prevalence rates to range between 4.1% to 9.3% in Taiwanese women and men aged 65 or older, respectively [23]. These differences could be heavily influenced by population differences as well as the variation in the cut-off values for sarcopenia noted between each study.

Amongst non-Asian populations, research in Brazilians aged 60 years or over has revealed an average overall prevalence of sarcopenia 17%, with rates of 20% in women and 12% in men [24]. In a study of individuals aged 65 years or older in Belgium, sarcopenia was reported to range from 9.25% to 18%, with the differences noted caused by variation in the cut-off values that were used in this specific study [25]. In the Maastrict study from the Netherlands, approximately 23% of adults 65 years or older were sarcopenic [26], and in the USA, in adults 60 years of age or older, prevalence is as high as 36.5% [27]. Additionally, it is reported that the prevalence of sarcopenia is 6.2% of men and 9% of women aged 65 years in Australia [28]. Despite available records, data for nominated Australia sub-populations, including Indigenous communities or for that matter indigenous groups in other national populations, are absent. Therefore, studies are required to provide a comprehensive understanding of sarcopenia prevalence in these and other groups, with the goal of developing effective targeted public health prevention and management strategies.

In addition, to the overall differences in sarcopenia prevalence noted above between men and women, a study by Kirchengast and Huber noted a higher prevalence of sarcopenia in women aged less than 70 years (females 31% vs. males 18.2%), but higher prevalence of sarcopenia in males aged over 80 years (males 50% vs. females 43.8%) [29]. This was suggested to relate to the more rapid decrease in steroid hormones that are responsible for the maintenance of muscle mass, in post-menopausal females. However, after the age 80, testosterone concentrations in males decline, which leads to a greater reduction in muscle mass in men of this age group, when compared to females [29].

## Mechanism of sarcopenia

### The genetics of sarcopenia

Studies to date have explored the relationship between Single Nucleotide Polymorphisms (SNPs) and sarcopenia, focusing on vitamin D receptor (*VDR*), interleukin-6 (*IL6*), alpha-actinin-3 (*ACTN3*), and Myostatin (*MSTN*) polymorphisms [30–34]. These revealed an association between *ACTN3* and *VDR* gene variants and sarcopenia, while no such association was observed for *IL6* variants and a specific *MSTN* variant linked to strength in athletes [30–35]. It is worth noting that these studies, employed both low muscle mass and impaired muscle function as criteria for defining sarcopenia. Other SNPs associating with sarcopenia include, fat mass and obesity associated gene (*FTO*) rs9939609 from fat, sex hormone Estrogen receptor 1 gene (*ESR1*) rs4870044, nitric oxide synthase 3 gene (*NOS3*) rs1799983 in the vascular endothelium, and Thyrotropin-releasing hormone receptor (*TRHR*) rs783255 [36]. Telomere attrition is another important mechanism to consider during ageing-associated sarcopenia progression [37]. For example, in older Chinese adults, longer telomeres have been linked to a slower decline in grip strength [38]. Moreover, a recent study has shown an association between shorter telomere length and increased sarcopenia incidence and persistence on older adults [39].

### The epigenetics of sarcopenia

Epigenetic modifications are widely recognized as significant regulators of skeletal muscle mass and repair [40]. Alterations in DNA methylation patterns and the presence of specific microRNA species are factors linked to age-related dysfunction in skeletal muscles, including sarcopenia [40]. Findings from the Hertfordshire Sarcopenia Study (HSS) show that the methylation changes associated with sarcopenia are concentrated in genes related to myotube fusion, oxidative phosphorylation, and voltage-gated calcium channels [41]. Moreover, it was observed that treatment of human primary myoblasts with GSK343, a histone methyltransferase Enhancer of Zeste Homolog 2 (*EZH2*) inhibitor, led to increased expression of the paired box transcription factor *PAX7*, which was associated with impaired myotube fusion and increased production of ATP. Treatment with GSK343 further altered the methylation status of genes linked to muscle energy generation (oxidative phosphorylation) and muscle growth (myogenesis) [41].

## Biomarkers in sarcopenia: a complex interplay of mechanisms

The progression of sarcopenia is principally associated with a series of changes, including neuromuscular junction modifications, changes in the endocrine system, altered growth factor expression, increased muscle protein turnover and changes in physiological behaviour. Consequently, it would be highly improbable that a single biomarker gene or protein could accurately and precisely determine the presence of sarcopenia in patients. To understand the underlying mechanisms more completely and to accurately identify elderly individuals with sarcopenia, it is important to develop a key panel of precise biomarkers for these pathways. Specific biomarkers are essential for clinical assessment to enable the identification of individuals with sarcopenia, or at risk of developing sarcopenia, and to monitor the effectiveness of prevention and treatment strategies. These biomarkers can be categorized according to different pathophysiological mechanisms associated with sarcopenia, which are outlined below.

### The neuromuscular junction (NMJ)

Sarcopenia pathogenesis frequently involves neuromuscular junction (NMJ) dysfunction. NMJs are crucial for transmitting muscle action potentials, and their malfunction can lead to neuromuscular fatigue, limiting exercise in individuals, especially the elderly [42–44]. Studies have suggested that NMJ dysfunction may result from increased proteolytic cleavage of agrin, which stabilizes the acetylcholine receptor (AChR). This cleavage produces a C-terminal agrin fragment (CAF) and is measurable in the serum. Elevated CAF levels associate with sarcopenia and neuromuscular fatigue, and correlate with loss of lean mass in the elderly [45]. Further markers of NMJ stability, notably brain-derived neurotrophic factor (BDNF), and glial cell line-derived neurotrophic factor (GDNF) have been assessed and a reduction in the levels of both have been associated with muscle loss and sarcopenia in Parkinson’s disease patients [46].

### The endocrine system

Hormonal imbalances play a significant role in sarcopenia. Essential hormones include growth hormone (GH), insulin-like growth factor-1 (IGF-1), dehydroepiandrosterone (DHEA), and testosterone. GH levels decrease with age, leading to lower daily secretion when compared to young adults [47]. The growth-promoting effects of GH are mediated through IGF-1, a hormone that stimulates muscle growth and regeneration, which is also decreased in sarcopenia [48, 49]. The levels of the adrenal steroid DHEA decline with age, influences muscle growth via IGF-1 and has varying associations with muscle strength [50]. Testosterone shows properties that counteract muscle breakdown, including reducing inflammation, and promoting muscle growth [51, 52]. Importantly, testosterone supplementation can prevent the reduced muscle mass and impaired muscle strength observed in elderly men, however, there are potential risks to treating with high doses of testosterone [53].

The levels of inflammatory proteins and cytokines are further altered during sarcopenia and an overall status of increased systemic inflammation in the body is associated with reduced muscle mass and impaired muscle function [54]. The levels of the inflammatory marker C-reactive peptide (CRP) have been shown to increase in circulation in patients with sarcopenia [55, 56]. Furthermore, elevated levels of the inflammatory cytokines, TNF-alpha and IL6, are associated with reduced muscle mass, muscle function and sarcopenia [56–58].

### Growth factors

Sarcopenia associates with an imbalance between factors that enhance and suppress muscle cell growth. In this regard, the transforming growth factor-β (TGFβ) superfamily member myostatin (GDF8), a potent negative muscle growth regulator, has received interest [59]. Some studies have reported elevated serum levels of myostatin in older individuals, however, the results are contradictory [60–63]. However, elevated levels of MSTN have been linked to decreased muscle function during aging [64, 65]. In addition, treatment with follistatin (FSTN), a myostatin inhibitor, has been shown to significantly increase muscle mass [66] and levels of FSTN have been shown to decrease during aging [67]. Further TGFβ superfamily members Activin A and Activin B have been implicated in muscle mass regulation, and in some conditions these growth factors have shown a more significant role in promoting muscle wasting than myostatin [68]. Levels of Growth Differentiation Factor-15 (GDF15), also a TGFβ superfamily member, are positively associated with aging and sarcopenia, but negatively associated with muscle mass [69, 70], supporting GDF-15 as a potential important biomarker of sarcopenia. The secreted pro-myogenic growth factor Irisin, has also been shown to play important roles in muscle growth, differentiation, repair, and regeneration [71, 72]. Additionally, the levels of Irisin are decreased during ageing and delivery of Irisin protein has been shown to improve sarcopenia in mice [73], providing evidence to support the importance of Irisin during sarcopenia.

### Muscle protein turnover

Early structural changes in skeletal muscle associated with sarcopenia are detectable through measuring distinct serum markers. The turnover of collagen type VI, and subsequent production of the degradation fragment (C6M) and type VI collagen N-terminal globular domain epitope (IC6), may serve as biomarkers of muscle mass [74]. However, the utility of these biomarkers in assessing muscle mass in aged individuals remains to be clarified. 3-Methylhistidine (3MH), a methylated form of histidine specific to myofibrillar proteins (e.g., myosin and actin), is another molecule associated with muscle proteolysis and assessment of serum levels of 3MH has been used to assess breakdown of muscle [75]. However, 3MH is also found in cardiac and smooth muscle tissue, so analysis of 3MH will not provide a specific measure of skeletal muscle protein breakdown alone [76]. Elevated levels of the skeletal-muscle isoform of Troponin T (sTnT), an important component of the muscle contractile unit, can also signify muscle damage [77]. Additionally, serum creatinine, a marker of muscle mass, has been used in conjunction with cystatin c (a protein produced by all nucleated cells) to create the sarcopenia index for assessing muscle mass [78]. However, further work needs to be done to validate the accuracy of this approach in diagnosing sarcopenia [79].

## Disease-related factors

### Hypertension and sarcopenia

Given the ever-increasing aging population and the rising prevalence of sarcopenia and hypertension (HTN), exploring the relationship between these two conditions has been considered. Several studies have provided a clear link between sarcopenia, elevated blood pressure and hypertension (HTN) [80, 81]. In agreement, a correlation between reduced muscle mass/strength and hypertension has been reported [82, 83], which is associated with increased risk of sarcopenia in older individuals [84]. Sarcopenia is associated with a chronic inflammatory state in the body, which is often linked to obesity, or what is called sarcopenic obesity, and insulin resistance (IR) [85]. Mechanistically, this chronic inflammation can 1) trigger the release of catabolic cytokines that are responsible for the breakdown of muscle proteins [85] and 2) stimulate the downstream renin-angiotensin-aldosterone system (RAAS), where overactivation of RAAS leads to the development of HTN [86]. Moreover, there is a defined relationship between IR, increased activity of RAAS and HTN [87, 88], which will further contribute to the reduced insulin response and increased HTN commonly observed in sarcopenic patients.

### Diabetes mellitus and sarcopenia

The risk of sarcopenia in Type 2 Diabetes Mellitus (T2DM) patients is greater when compared to non-diabetic individuals [89]. T2DM and Sarcopenia exhibit a bidirectional association [90], with the presence of each increasing the risk of the other [91]. The main pathology associated with T2DM is IR. Skeletal muscle, as a peripheral tissue, is a major target insulin action in the body and importantly, impaired insulin action promotes protein degradation, leading to reduced skeletal muscle mass and strength [92]. Mitochondrial dysfunction is a common comorbidity of sarcopenia and is fundamentally linked to decreased muscle mass and function [93] and development of insulin resistance [94]. Mitochondrial dysfunction in diabetics results in impaired lipid oxidation and IR, leading to increased lipids in muscle cells and the development of sarcopenia [95]. Furthermore, loss of muscle mass, secondary to age and sarcopenia, causes metabolic dysregulation leading to decreased insulin sensitivity, changed oxidative defences, and impaired mitochondrial function [96]. In addition, testosterone and IGF1, which are hormones involved in muscle protein synthesis, are decreased in patients with T2DM [97, 98].

### Heart failure and sarcopenia

Altered muscle composition and function are important factors associated with heart failure progression [99]. Importantly, a common catabolic response and loss of myofibrillar proteins is noted in skeletal muscles during chronic heart failure [100]. Sarcopenia can cause a poor prognosis in heart failure patients, and a recent systematic review noted that 34% of patients suffering from heart failure also had sarcopenia [101]. Heart failure patients on medications, such as digoxin and diuretics, are prone to develop nausea and gastroenteropathy, which ultimately causes anorexia, malabsorption, weight loss and eventual loss of muscle mass and strength [102, 103], potentially exacerbating the sarcopenic phenotype. In addition, physical inactivity, which is common in heart failure patients, can cause decreased insulin sensitivity which can adversely affect muscle metabolism [104]. In agreement, heart failure is commonly associated with IR [105]. Moreover, low physical activity can impair mammalian target of rapamycin (mTORC1) signaling, which interferes with muscle protein synthesis and skeletal muscle growth [106].

### Coronary artery disease and sarcopenia

Coronary Artery Disease (CAD) is the most common form of CVD, with HTN and T2DM being important risk factors for the occurrence of CAD [107]. Handgrip strength is known to be an independent predictor of CAD [108], and an inverse association between muscle mass and CAD has been reported [109]. One potential mechanism to explain this relationship is that lower muscle mass may reduce whole-body energy expenditure, leading to fat accumulation and a greater risk of CAD.

**Figure 1:**
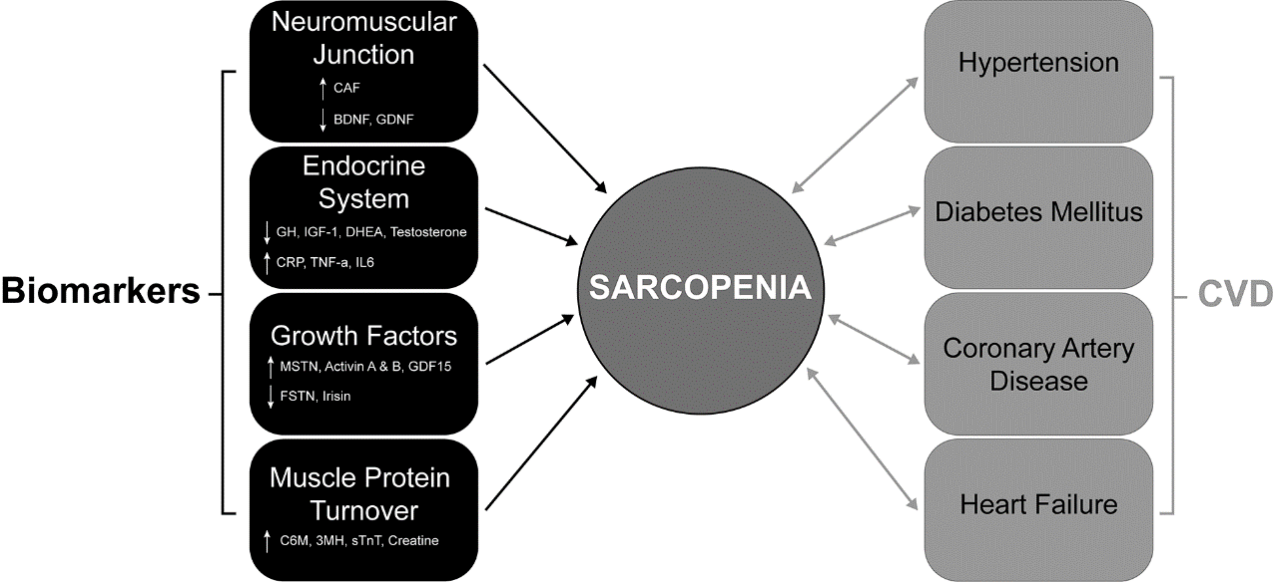
**The bidirectional association of CVDs and their comorbidities with sarcopenia.** Diagram showing the complex interactions that contribute to sarcopenia development in CVDs (coronary artery disease and heart failure) and their comorbidities (hypertension and diabetes mellitus). Significant changes in key proteins (circulating biomarkers) associated with muscle function, neuromuscular junction, protein turnover and the endocrine system contribute to sarcopenia. ↑ = Increase and ↓ = decrease. CVDs and their comorbidities all contribute to sarcopenia and importantly, this relationship is bidirectional, with the presence of sarcopenia also contributing to the CVD comorbidities. CVD; Cardiovascular Disease.

Studies have examined sarcopenia as a predictive factor for poor major adverse cardiovascular event (MACE) outcomes in patients with CAD [110]. This can be explained in part due to altered expression and secretion of muscle-specific growth factors (Myokines) from sarcopenic muscle. The levels of specific myokines are variable in patients with heart failure and CAD. Specifically, the levels of the promyogenic factor Irisin are decreased in CAD [111], which is important as Irisin has been shown to have several cardiac protection functions in pre-clinical models [112]. In contrast, levels of the inhibitory myokine Myostatin are increased in heart failure patients [111], where overexpression of Myostatin in the myocardium is associated with increased fibrosis in the heart [113]. The myokine BDNF is primarily cardioprotective, with lower levels of BDNF observed in CAD patients [114]. An overview of sarcopenia biomarkers and interactions between cardiovascular disease risk factors and sarcopenia is summarized in Figure 1.

### Cardiac surgery and sarcopenia

Cardiac surgery is becoming prevalent in more complex cases that include elderly frail patients who have multiple comorbidities, including sarcopenia. In addition, research has shown that patients with characteristics of sarcopenia are at increased risk of postoperative complications [115–117]. Cardiopulmonary bypass (CPB) is a technique that temporarily takes over heart and lung function during cardiac surgery. Controversy relating to the duration of CPB in sarcopenic and non-sarcopenic patients has been reported, with some studies revealing a prolonged CPB time in sarcopenic patients, while others reported no effect of sarcopenia on the duration of CPB [118–120]. In addition, intubation times for Sarcopenic patients are increased when compared to non-sarcopenic patients, which can be explained by diminished respiratory muscle function and lower physiological reserve, which is the capacity for cells, tissues and organs to function beyond their baseline level [7]. As a result these patients are more prone to complications and longer stays in intensive care units and display higher rates of early and late mortality post cardiac surgery [7]. Generally, sarcopenia is reported to also be a significant predictor of mortality in other major surgeries [8]. Nonetheless, despite the increased mortality rate in patients with sarcopenia, no significant difference in the risks of surgical wound infection, arrhythmia, and stroke, following cardiac surgery in sarcopenic patients has been reported [7].

Furthermore, the presence of sarcopenia has a significant effect on the duration of hospital stay, discharge transfers and cardiac rehabilitation [120, 121]. This may be due to the decreased ability of these patients to tolerate the physiological stress of surgery, which results in a slower recovery. Thus, cardiac rehabilitation programs may be critical in meeting the challenges associated with sarcopenia. Moreover, involving multiple professional specialisations, including physicians, physiotherapists, and nutritionists during cardiac rehabilitation has been shown to significantly improve the outcomes of patients undergoing cardiac surgery [122]. In the future it will be important to consider how identification of sarcopenic patients and subsequent intervention in the form of rehabilitation prior to surgery could potentially prevent and manage the associated complications.

Importantly, several risk models, including the European System for Cardiac Operative Risk Evaluation, EuroSCORE II [123] and the Society of Thoracic Surgeons predicted Risk of Morbidity and Mortality (STS-PROMM) [124] have introduced risk scoring in cardiac surgery. However, these risk models focus on medical comorbidities and do not include the functional status of frailty or sarcopenia. Given the association of sarcopenia with poorer health and surgical outcomes, consideration of sarcopenia status may be important for future iterations of these risk scoring systems for cardiac surgery.

## Screening

Detecting sarcopenia before any apparent symptoms can minimize the risk of severe consequences. As such, screening questionnaires have been created and trialled for this purpose. Several years ago, Malmstrom and Morley et al., introduced the SARC-F questionnaire [125]. SARC-F is a questionnaire considering: Strength (S), Assistance walking (A), Rising from a chair (R), Climbing stairs (C), and Falls (F) on a scale of 0 to 2 for each component (see Table 2 for details). SARC-F has been a popular screening tool for sarcopenia, however, studies have reported SARC-F to be highly specific but have low sensitivity [126]. More recently, Barbosa-Solva et al., introduced a modified questionnaire, that included calf circumference (CC) as an additional measurement, called SARC-CalF (SARC-F combined with Calf Circumference), and this was reported to be a highly sensitive and specific questionnaire [127]. An additional questionnaire, the mini sarcopenia risk assessment (MSRA), which is available in short (MSRA-5) and full (MSRA-7) versions has been introduced, but there is still a lack of evidence to support the use of this questionnaire to date [128].

**Table 2:** Screening tools for sarcopenia.

**Questionnaire**	**Domains and specific questions**	**Comments**
**SARC-F [125]**	**Muscle Strength:** Q: How much difficulty do you have in lifting and carrying 10 pounds (4.5 kg)? (score: none = 0, some = 1, a lot or unable = 2) **Ambulation:** Q: How much difficulty do you have walking across a room? (score: none = 0, some = 1, a lot or unable without help = 2) **Chair rise:** Q: How much difficulty do you have transferring from a chair or bed? (score: none = 0, some = 1, a lot or unable without help = 2) **Stair Climbing:** Q: How much difficulty do you have climbing a flight of 10 stairs? (score: none = 0, some = 1, a lot or unable without help = 2) **Falls:** Q: How many times have you fallen in the past year? (score: none = 0, 1-3 falls = 1, 4 or more falls = 2) **Positive if score ≥ 4**	**Advantages:** Highly specific Self-reporting Quick **Disadvantage:** Low Sensitivity
**SARC-CalF** **(SARC-F + Calf Circumference) [127]**	Questions and scoring for the first 5 domains are identical to SARC-F above: Muscle Strength (score 0-2) Ambulation (score 0-2) Chair rise (score 0-2) Stair Climbing (score 0-2) Falls (score 0-2) The new component is measurement of Calf Circumference (CC): Measure the patient's exposed right CC with the legs relaxed and feet 20 cm apart from each other (score 0 or 10) Score 10: CC ≤ 33 cm in female Score 10: CC ≤ 34 cm in male **Positive if score ≥ 11**	**Advantages:** Highly specific Highly sensitive
**MSRA 5 [128]**	Age (score ≥70 years = 0 or <70 years = 5) Hospitalisation history in last year (score: >once = 0, once = 10 or none = 15) Physical activity capacity (score: walk <1000m = 0 or walk >1000m = 15) Three meals a day (score: no = 0, yes = 15) Weight Loss in the last year (score >2kg = 0 or ≤2kg = 10) **Positive if score ≤ 45**	**Advantage:** Higher Specificity than MSRA 7 **Disadvantages:** Lower Sensitivity than MRSA 7 Limited use in cardiac patients, given questionnaire validated only in NYHA class 0-1 patients, that have normal kidney function and no cognitive impairment.
**MSRA 7 [128]**	Age (score ≥70 years = 0 or <70 years = 5) Hospitalisation history in last year (score: >once = 0, once = 5 or none = 10) Physical activity capacity (score: walk <1000m = 0 or walk >1000m = 5) Three meals a day (score: no = 0, yes = 5) Food consumption 1, milk or dairy products (score: not every day = 0, at least once a day = 5) Food consumption 2, protein (score: not every day = 0, at least once a day = 5) Weight Loss in the last year (score >2kg = 0 or ≤2kg = 5) **Positive if score** ≤ **30**	**Advantage:** Higher Sensitivity than MRSA 5 **Disadvantages:** Lower Specificity than MRSA 5 Limited use in cardiac patients, given questionnaire validated only in NYHA class 0-1 patients, that have normal kidney function and no cognitive impairment.

Table summarising current screening tools for sarcopenia. SARC-F, Strength, Assistance walking, Rise from a chair, Climb Stairs, and Falls; SARC-CalF: Strength, Assistance Walking, Rise from a chair, Climb Stairs, Calf circumference and Falls; MSRA: Mini Sarcopenia Risk Assessment questionnaire; NYHA, New York Heart Association.

According to the EWGSOP2 definition, sarcopenia is represented by loss of muscle mass with clinical repercussions, in other words a loss of muscle function. The proposed SARC-CalF predicts subjects to be positive for sarcopenia when they display a total of 11 points, which is comprised of low muscle mass (10 points) and a minimum of one symptom of muscle function loss (1 to 10 points) [127]. Participants who present symptoms of muscle function loss without low muscle mass, do not under the EWGSOP2 definition have sarcopenia and are also not identified as having sarcopenia according to SARC-CalF.

The study by Barbosa-Solva et al. also compared SARC-F with SARC-CalF and used the receiver operating characteristic (ROC) curve and considered the area under the curve (AUC) for this comparison [127]. The SARC-F performance as a screening tool for sarcopenia showed an AUC of 0.592, which suggested insufficient sensitivity for detecting sarcopenia. However, the inclusion of CC with SARC-F improved the questionnaire sensitivity 2-fold (from 33% to 66%) without compromising its specificity with an AUC of 0.736 [127]. A further study by Krzymińska-Siemaszko et al. also supported the use of SARC-CalF when validated against all diagnostic criteria with a sensitivity as high as 75% and an AUC ranging between 0.711-0.874 [129].

It has been reported that a limitation in CC assessment is the influence of intramuscular or subcutaneous adipose tissue deposition and peripheral oedemas [130, 131], as a result, obese patients can show a high CC. As increased adiposity is a common comorbidity of sarcopenia, which is referred to as sarcopenic obesity [132], questionnaires alone may be inadequate for screening obese patients for sarcopenia with a more formal diagnostic evaluation required.

A concise summary of the four common screening questionnaire tools is provided in Table 2.

## Imaging in sarcopenia

A wide spectrum of radiological imaging modalities, including dual-energy X-ray absorptiometry (DXA), computed tomography (CT), magnetic resonance (MR), and ultrasound (US), are used to assess muscle quality and mass to facilitate the “clinical” diagnosis of sarcopenia. A summary of the imaging modalities used for sarcopenia and their benefits and limitations is provided in Table 3.

**Table 3:** Imaging tools for sarcopenia.

**Tool**	**Measurement**	**Advantages**	**Disadvantages**
**Dual-energy X-ray absorptiometry (DXA) [133–135]**	Appendicular and whole body lean mass	Inexpensive Widely Available Fast Acquisition Low Radiation Risk Cut-off values available Simultaneous measurement of whole-body fat mass and bone mass Highly recommended by EWGSOP	2-dimentional data Not including trunk muscles No muscle quality data Confounded by oedema and obesity
**Computer Tomography (CT) [136, 137]**	Cross sectional area of individual or group of muscles Attenuation values	Measures muscle mass and muscle quality Numerous indications allow opportunistic use Highly accurate Differentiate between fat and fat free mass	Expensive High Radiation Risk No cut-off values Low Availability Time consuming segmentation process Commonly used at L3 level, which has low opportunistic utility in cardiac conditions.
**Magnetic Resonance Imaging (MRI) [137, 144]**	Cross-sectional area of individual or group of muscles Fat content by Dixon imaging	Measures muscle mass and muscle quality Highly accurate Best spatial resolution Body mass composition differentiation No Radiation Risk	Expensive No cut-off values Low Availability Long acquisition time
**Ultrasound (US) [145–148]**	Cross-sectional area Muscle thickness and echo-intensity	Measures muscle mass and muscle quality Inexpensive Widely available Fast Acquisition No radiation Real time visualization of target structure	Operator dependent No cut-off points Poor accuracy Scarcely reproducible

Table summarising current imaging modalities used to assess skeletal muscle mass and clinically diagnose sarcopenia. Important advantages and disadvantages for each approach are listed. Dual-energy X-ray absorptiometry (DXA), Computer Tomography (CT), Magnetic Resonance Imaging (MRI), Ultrasound (US).

### Dual-energy X-ray absorptiometry (DXA)

DXA is the most commonly used radiological tool, employing an x-ray source to evaluate body composition. This allows the concurrent measurement of lean mass (LM), fat mass (FM), and bone mineral content (BMC). LM evaluation can estimate all non-fat/non-bone tissues [133]. The appendicular lean mass (ALM) value (the sum of LM measurements from lower and upper limbs) is the calculated measurement for muscle mass obtained from DXA scans, where the ALM is then indexed to height, to calculate the ALM index (ALMI = ALM/height^2^), which forms an important measure for sarcopenia [134]. The new EWGSOP guidelines have slightly modified prior diagnostic cut-off values, recommending an ALMI of <5.5 kg/m^2^ in females and an ALMI of <7.0 kg/m^2^ in males to define low muscle mass and confirm the presence of sarcopenia [13]. DXA cannot assess muscle quality (muscle fat infiltration), and its measurements are affected by the hydration status of patients [134, 135]. However, clinically, the benefits of DXA outweigh the limitations. Thus, the EWGSOP guidelines suggest that DXA be used as the first tool for clinical assessment of sarcopenia, with MR and CT-based approaches more suited to research studies [13].

### Computer tomography (CT)

Computer Tomography (CT) can be used to assess both muscle mass, muscle density and muscle quality (muscle fat) [136]. CT can distinguish lower density fat pixels from higher density muscle pixels, which allows the effective subtraction of intramuscular fat from muscle mass. In contrast, DXA offers estimation of whole-body lean mass, while CT can measure muscle attenuation (density) and size in distinct regions [136]. Measurements obtained from a single cross-sectional area (CSA) of a CT slice are very accurate in estimating body composition, and there is a strong association between skeletal muscle distribution, whole-body and single-slice adipose tissue [137]. Notably, CSA is not commonly applied alone but indexed for height (CSA/height^2^) to obtain the Skeletal muscle index (SMI) for muscle mass. Some sarcopenia studies have used thigh CT analysis to classify sarcopenia [138, 139]. In addition, the pectoralis major muscle has also been identified as another target muscle for confirming sarcopenia [140]. However, abdominal CT imaging of the psoas muscle at L3 or L4 level, appears to be the preferred method for evaluating sarcopenia [141]. Notably, recent work has revealed that analysis of only the psoas muscle at these single points may not be the best approach in all conditions, with analysis of all muscles at L3 suggested as a more reliable analysis [142].

CT has the advantage of being routinely utilised for staging in several conditions, such as during cancer follow-ups and during pre-cardiac surgery assessment and is therefore optimal for opportunistic assessment of sarcopenia without the need of additional examinations. However, no consensus is currently available for standardized CT thresholds to diagnose sarcopenia. Nonetheless, CT is currently used for research purposes in several retrospective and prospective analyses [143].

### Magnetic resonance imaging (MRI)

MRI can measure the amount of fat and muscle because of its multipara-metricity and high contrast resolution. Like CT, MRI has a very high accuracy in evaluating fat and muscle volumes [144] and has been shown to be a useful tool for estimating skeletal muscle mass [137]. MRI has the potential to be an optimal tool for sarcopenia imaging as it has no radiation exposure. However, it is mainly used for research and cannot be effectively used in clinical practice because of its long scan and post-processing time, high expense, and lack of current protocol standardization.

### Ultrasonography

Ultrasound (US) scans can be used to reliably assess muscle quality and mass and have potential for sarcopenia [145]. It has high inter-reader reliability to evaluate muscle CSA and shows no significant difference to that of MRI in muscle CSA and volume measurement [146]. Ultrasound is highly utilised for several musculoskeletal diseases and is readily available and cost effective [147]. However, despite the potential for US in diagnosing sarcopenia, there are currently no defined cut-off values for muscle mass loss and muscle quality parameters, which limits its use for sarcopenia in clinic [148].

## Treatment of sarcopenia

Sarcopenia management and treatment should be based on a thorough understanding of its pathophysiology. Importantly, both pharmacological and non-pharmacological approaches have been considered for treating sarcopenia, a summary of which is provided in Table 4.

**Table 4:** Sarcopenia treatments and interventions.

**Modality**	**Intervention (with reference)**	**Effect**	**Notes / Comments**	
**Nutrition**	High-protein diet + prolonged resistance exercise [151]	↑ muscle mass ↑ muscle strength	Synergistic effect is observed when both interventions are combined. Conflicting results across different studies have been noted.	
	BCAAs, especially. Leucine [152–154]	↑ protein synthesis ↑ muscle mass (some functional gain)	Soluble protein forms are most effective. Studies tended to be in healthy older individuals. The presence of chronic illness, such as heart disease and cancer, was typically an exclusion criterion. More studies looking at benefit in patients with chronic illness should be undertaken.	
	Vitamin D alone [159, 160]	Only minimal ↑ in muscle strength no effect on muscle mass	Studied in elderly and postmenopausal women. More work needs to be done to understand the function of Vitamin D in sarcopenia.	
	Vitamin D + Leucine-enriched protein [161]	↑ muscle mass & function (e.g., chair, sit-stand capability)	Synergistic supplementation in elderly with mild to moderate mobility limitations. Patients with comorbidities excluded, such as kidney or liver failure.	
**Exercise**	Aerobic exercise (walking, cycling) [167–169]	↑ muscle CSA ↑ muscle quality (defined as strength per unit muscle mass) ↓ blood pressure ↑ capillary density	Cardiovascular and metabolic benefits	No change in lean muscle mass. Less hypertrophic effect than resistance training. May not be viable for patients with functional decline or frailty, patients with chronic diseases, such as CVD were excluded.
	Resistance exercise [171, 172]	↑ muscle mass, strength and function	Primary non-pharmacological approach	No standardised program developed. Patients with chronic diseases may require different management approaches.
	Exercise frequency & duration [173–175]	1-2 session(s)/week can help ↑ muscle strength ≥3 months required for significant clinical effect: e.g., a significant ↑ muscle strength and physical performance.	Systematic review supports long-term benefit	Lack of standardisation and universal guidelines
**Pharmacological**	Testosterone [177–183]	↑ muscle mass ↑ muscle strength	Effective in hypogonadal men	Limited clinical trial data. No benefit on physical performance (muscle function); not recommended in patients without hypogonadism (limited use). Can lead to polycythemia and sleep apnoea, which negatively impacts patients with reduced cardiac reserve. Increases risk of cardiovascular events.
	SARMs [184–187]	↑ muscle and bone mass in pre-clinical rodent models ↑ muscle mass and physical function in clinic	Activates androgen receptor (AR) signalling	Potential hepatotoxicity effects [188, 189]
	Estrogen/hormone replacement therapy (HRT) [190–192]	Conflicting studies. Beneficial effects on muscle mass & strength in postmenopausal women have been shown. However, studies have also shown no effect on muscle mass and strength.	Used in estrogen-deficient women	Dose and physical activity of person important for beneficial effects.
	Growth Hormone (GH) [193–200]	↑ muscle mass Some evidence of improved muscle strength ↓ fat mass	Stimulates IGF-1 signalling. Increases metabolism	↑ Insulin resistance, diabetes and metabolic syndrome. Increased risk of cardiovascular events. Unclear muscle function improvement.
	ACE inhibitors / ARBs [205–209]	Some benefit for improving muscle mass and muscle function	Cardiovascular medications with secondary muscle benefits	However, conflicting evidence is noted: benefits of ACE inhibitors on muscle mass, function and performance are inconsistent.
	Ruvembri™ (Sarconeos / BIO101) [210–213]	↑ protein synthesis in mouse C2C12 myotubes and human primary myotubes ↑ myoblast differentiation (mouse C2C12 cells) ↑ Physical performance in pre-clinical mouse models ↑ gait speed in phase 2 clinical trial	Activator of MasR (a receptor of the renin–angiotensin system); shown effective in Phase 2 (SARA-INT trial)	Good safety and pharmacokinetic profile. Phase-2 clinical trial, shown to be effective for improving gait speed. Phase 3 protocol prepared.
	Vorinostat (HDAC inhibitor) [215]	Studies using mouse C2C12 muscle cells have shown that Vorinostat: ↑ myotube size (diameter), ↑ muscle cell differentiation ↓ myoblast proliferation	Only *in vitro* results (C2C12 muscle cells). Seems to inhibit proliferation to promote differentiation of muscle cells (cell cycle regulation)	No clinical trials yet, requires rigorous pre-clinical and clinical validation. Already FDA approved for T-cell lymphoma. As Vorinostat is an HDAC inhibitor off-target effects, including cardiac effects, can be an issue [216].

Summary of pharmacological and non-pharmacological treatment options for sarcopenia. Relevant references for each treatment modality along with known effects and important considerations are included. ↑ = Increase and ↓ = decrease. AR, Androgen Receptor; ACE, Angiotensin-Converting Enzyme; ARB, Angiotensin Receptor Blocker; BCAAs, Branched-Chain Amino Acids; CSA, Cross Sectional Area; FDA, United States Food and Drug Administration; HDAC, Histone Deacetylases; HRT, Hormone Replacement Therapy; MasR, Mas Receptor; SARA-INT trial, Sarcopenia-Interventional trial; SARM, Selective androgen receptor modulators.

To date, treatment options for sarcopenia in clinical practice include nutritional supplementation and resistance training [149, 150], which links with decreased patient hospitalization through increases in muscle trophism and strength [150].

### Nutrition

Although the effectiveness of nutritional intervention without exercise is not known in sarcopenia management, some dietary patterns, including adequate intake of vitamin D, protein, antioxidants, and long-chain polyunsaturated fatty acids have been reported to be effective [149]. Moreover, a combination of high-protein diet and resistance exercise is described to increase muscle strength [151]. Branched chain amino acids including the essential amino acid Leucine are important for protein synthesis in the body [152]. Importantly, soluble Leucine-enriched protein supplementation, is effective in enhancing muscle mass and to a lesser extent muscle function in older individuals [153, 154]. Although protein intake requirements will most likely vary between individuals and different populations around the world, recommendations from the European Society for Clinical Nutrition and Metabolism (ESPEN) expert group suggest between 1.0-1.2g protein/kg/day for healthy individuals and between 1.2-1.5g protein/kg/day protein for individuals with chronic illness [155]. Whereas the US Food and Nutrition Board (FNB) recommends protein intakes for all adults of 0.8g protein/kg/day [156].

The “Mediterranean diet” is known to be rich in nutrients has been associated with improved muscle mass and function [157] and has important health benefits for individuals with coronary heart disease [158].

Vitamin D effectiveness has also been investigated for potential benefits on muscle mass, muscle strength and physical performance. Intriguingly, vitamin D supplementation has been shown to only have a minimal effect on muscle strength and no effect on muscle mass in elderly [159, 160]. However, supplementation of diet with both protein (leucine-enriched) and vitamin D can improve muscle function, including stair climbing ability, and increase muscle mass in older adults with sarcopenia [161].

### Exercise

Lack of exercise and inactivity are important risk factors for sarcopenia [162]. Physical exercise, including aerobic and resistance training, can be a safe and effective intervention for sarcopenia and has proven utility for increasing muscle mass, strength, and function [163–166].

Aerobic exercise, including cycling and walking, has been shown to reduce blood pressure in patients with resistant hypertension [167], which has important implications for overall cardiovascular health. Aerobic exercise has been shown to increase muscle CSA and improve muscle quality [168, 169] but is less effective than resistance exercise at promoting skeletal muscle hypertrophy than resistance exercise [170].

Resistance exercise is the primary non-pharmacological treatment for sarcopenia increasing muscle mass, strength, function [171] and has important benefits for patients with heart failure [172]. Although the importance of resistance exercise is clear, no exercise regime specifically designed to treat patients with sarcopenia has been developed, and at this stage the programs offered to sarcopenic patients can be quite variable [173]. However, reports suggest that as little as one exercise session per week has been shown to improve muscle strength in aged individuals [174]. However, results from a systematic review by Cruz-Jentoff et al., suggest that periods of at least 3-months of exercise interventions are required to significantly improve clinical readouts [175]. Mechanistically, resistance exercise is reported to suppress chronic inflammatory factors, decrease oxidative stress, and reduce pathways involved in protein breakdown, such as the ubiquitin proteasome-mediated protein degradation [176].

### Pharmacology

Presently the are no specific drugs approved by the Food and Drug Administration (USA) for sarcopenia treatment. However, research focusing on identifying and validating potential drug-based therapeutics for sarcopenia is well underway and the efficacy of several strategies and drugs will be discussed below.

Testosterone is an important determinant of muscle function and for muscle mass maintenance during aging [177]. Studies have reported testosterone supplementation to be effective for improving muscle mass and strength in older individuals with different levels of testosterone and hypogonadism severity [178–180]. Nonetheless, the effectiveness of testosterone in physical performance is negligible [177, 181]. Thus, testosterone treatment is not recommended to be used in the absence of clear symptoms of hypogonadism [182, 183].

Selective androgen receptor modulators (SARMs) are a class of synthetic androgen receptor (AR) ligands that are able to bind AR and activate downstream signalling [184]. Clinical studies utilising SARMs have shown promise related to improving muscle mass and strength in older individuals [185–187]. However, the safety of SARMs is questionable, with published reports identifying hepatotoxic effects [188, 189].

The risk of sarcopenia is higher in post-menopausal women and is related to declining estrogen levels [190, 191]. Importantly, studies have reported a beneficial effect of hormone replacement therapy (estrogen), and in increasing muscle strength, mass and performance in post-menopausal women [191, 192].

Growth hormone (GH), which is the main hormone stimulating the secretion of IGF-1, can promote the growth and development of organs and tissues, protein synthesis and it can affect fat, protein and carbohydrate metabolism [193]. Although GH has been shown to increase muscle mass, the benefits of GH supplementation on muscle function in older individuals are less clear [194–196]. Growth hormone can also increase the risk of insulin resistance and adversely affects the cardiovascular system [197–200]. Thus, there are concerns about the overall benefit of using GH supplements.

Reduced Insulin-like Growth Factor 1 (IGF-1) levels are associated with sarcopenia [48, 201] and moreover, administration of IGF-1 has been shown to promote protein synthesis [202, 203]. IGF-1 has been investigated for improving muscle strength and function in patients with spinal and bulbar muscular atrophy [204], however, no improvement in muscle strength or function was noted upon treatment with the IGF-1 mimetic (BVS857) [204].

Studies in older adults have revealed improved muscle function upon taking a class of drugs that lower blood pressure, including Angiotensin converting enzyme inhibitors (ACEis) and angiotensin receptor blockers (ARBs), which block angiotensin II production and function, respectively [205]. Importantly, these are acknowledged medications for the treatment of heart failure. However, studies have shown mixed results on the benefits of ACEis and ARBs in improving muscle mass and function [205–209], as such further work is required to further characterise the benefit of ACEis and ARBs in sarcopenia.

More recently, a series of drugs have been developed that are currently being tested in clinical trials for safety and efficacy in treating sarcopenia. Ruvembri™, also known as Sarconeos and BIO101, is based on a purified form of 20-hydroxyecdysone, which is a steroid hormone found in arthropods [210]. Ruvembri™ functions as a Mas receptor (MasR) activator in the renin-angiotensin system and has been shown to promote protein synthesis in muscle and improve muscle cell differentiation [211, 212]. A phase 2 clinical trial (SARA-INT) with Ruvembri™ have shown efficacy for treating sarcopenia with an increase in gait speed noted over a 400m walking test in older individuals [213].

Isomyosamine (MYMD-1) is a synthetic plant alkaloid that functions as a cytokine inhibitor targeting cytokines such as TNF-α [214]. Phase 1 clinical trials have been completed with decreased levels noted in healthy adults, with further clinical trials planned [214].

Vorinostat, a histone deacetylase inhibitor has also been identified [215], which has already been approved for treatment of haematological malignancies [216]. Current research has demonstrated the utility of Vorinostat in muscle cell differentiation, with increased myotube size noted upon treatment of C2C12 muscle cells [215].

Importantly, while several pharmacological interventions are under investigation including selective androgen receptor modulators (SARMs), growth hormone, and IGF-1 analogues many face hurdles in translation to clinical use due to inconsistent efficacy and safety concerns, such as hepatotoxicity and metabolic dysregulation. Although agents like Ruvembri™ (BIO101) have shown early promise in improving mobility metrics in phase II trials, no pharmacological therapy has yet gained approval for sarcopenia, and long-term outcome data remain sparse. These limitations emphasize the need for robust, large-scale clinical trials that assess both efficacy and safety across diverse populations.

## CONCLUSIONS

There are shared pathogeneses between sarcopenia and CVD which remain to be further studied. Validated screening questionnaires and definitional criteria that target specific populations are of great importance in detecting early-stage sarcopenia and thereby preventing occurrence and reducing severity. This will have the follow-on effect of improving the prognosis of cardiovascular disease in non-surgical and surgical stages of management. Although pharmacological approaches are being developed for sarcopenia, non-pharmacological interventions that include nutritional supplements and resistance exercise remain the gold-standard and are necessary steps to obviate the bi-directional relationship of sarcopenia with CVD. Pre-operative rehabilitation could be introduced in selective cases with sarcopenia to avoid major intra- and post-operative independent complications of sarcopenia in cardiac surgery. Moreover, including sarcopenia as a factor on EuroSCORE II and STS risk scoring systems would be important for monitoring patients.
